# Lower attention to daily environment: a novel cue for detecting chronic horses’ back pain?

**DOI:** 10.1038/srep20117

**Published:** 2016-01-29

**Authors:** C. Rochais, C. Fureix, C. Lesimple, M. Hausberger

**Affiliations:** 1 Université de Rennes 1, UMR CNRS 6552 -Laboratoire Ethologie Animale et Humaine-EthoS- Station biologique, 35380, Paimpont, France; 2 CNRS- UMR 6552 Université de Rennes 1, -Laboratoire Ethologie Animale et Humaine- 263 avenue du général Leclerc, 35042, Rennes cedex, France

## Abstract

Chronic pain is thought to affect patients’ cognitive functioning, including attention. Loss of attention is likely to have an impact on the execution of daily tasks, and, therefore, to have negative effects. However, relationships between chronic pain and cognitive deficits are still debated. Pre-clinical studies using laboratory animals prove useful to model pain-related cognitive impairment, but animal models had to predict effects in the real world. This study investigates attentional engagement of domestic horses by comparing observations in a home setting and evaluations of vertebral disorders. We found that lower attentional engagement and the level of back disorders were correlated. Two different evaluation techniques of the state of horses’ spines gave similar results. We suggest that novel animal models would prove useful for identifying spontaneous behaviours indicative of chronic pain. We suggest that more ethological studies in human patients’ home environments would help to improve our understanding of the processes involved. Finally, these results yield interesting indications for evaluating animal welfare, as attentional engagement could become a reliable indicator of chronic pain and thus a useful tool for identification of suffering individuals.

Chronic pain is thought to affect patients’ cognitive functioning. As the neural systems involved in cognition and pain processing are closely linked, they may modulate one another^1^. Chronic pain has an impact on a subset of cognitive domains including attention, learning, memory, or speed of information processing^2^. Loss of function in these domains is likely to have an impact on the execution of daily tasks and to affect relationships, capacity for work, moods, quality of life and, therefore, affect individuals negatively^3^.

An increasing body of evidence from anatomical, neurochemical, and behavioural studies supports the concept of structural and functional plasticity of the brain of patients with chronic pain (*i.e.* pain persisting for at least 3 months) (*e.g.*^4^,^5^,^6^). The chronic pain literature repeatedly reports attention deficits and other cognitive impairments of patients with chronic low back pain (CLBP), fibromyalgia, migraine, rheumatoid arthritis, and musculoskeletal pains (*e.g.*^7^,^8^,^9^). Pain is an inherently attention-demanding sensory process and distraction of attention has been shown to reduce perception of acute pain^10^. Although some studies report a direct correlation between the levels of cognitive dysfunction and pain ratings by patients^1^,^11^, relationships between chronic pain, in particular chronic low back pain, and cognitive deficits remain debated^11^,^12^. The effects of chronic pain on neuropsychological functions could be cumulative and quantitatively different from acute/experimental pain^6^. Different procedures, medication of patients with chronic pain, subjective perception of pain and experimental clinical settings (*i.e.* different from daily living conditions) are aspects that can explain controversial findings^1^.

Although pre-clinical studies using laboratory animals as models prove useful to model pain-related cognitive impairments^1^, studies of animals in their environment are still scarce. Difficulties of these studies are related to the fact that they are based on locomotor activity and appetitive responds, which can decrease as a result of the pain induced^1^. A report showed that rats’ vigilance decreased with increased arthritic pain even under transient analgesia and concluded that fundamental areas for attention processing were affected by the development of chronic pain^13^. Animals can thus provide interesting models of attention deficits in chronic pain. However, a model is a tool for predicting effects in the real world; a link must be maintained between the model and the system of interest^14^. Therefore, observations of unconditioned behaviour provide a rapid, economical and justified means to quantify animals’ attention^15^. For example, rats’ head scans (*i.e.* a rat elevates its head, moves it side to side, and lowers it) signal general alertness and have been used to assess the effects of toxin-induced lesions in the forebrain^16^. Rats that did not perform this behaviour were considered to express ‘sensory inattention’.

Humans can also express sensory inattention: because chronic pain is an environment where the source of pain cannot be removed, it can induce a chronic interruption of current attentional engagement, associated with a widespread avoidance of pain and movement and a withdrawal from social contacts^17^. Patients with chronic pain ‘inhabit an impoverished and restricted environment’^17^. This daily loss of attention paid to their environment is not assessed by clinical studies on pain and cognition and thus appears only through self-reports. The extent pain chronicity or intensity influences spontaneous daily attentional engagement remains unknown. This is where spontaneous animal models could be of great help.

Domestic animals (dogs, cats and horses) share their environment with humans (*e.g.* daily working activities) and, in common with humans, frequently suffer from neck or back pains (more than 60% North American humans, more than 70% riding horses in Europe^18^,^19^,^20^,^21^,^22^). Diagnoses are difficult for these species and many dogs, cats and horses are treated inappropriately or not at all if they do not show overt clinical signs^18^,^22^,^23^,^24^. If sensory inattention (*i.e.* attention deficit) is a behavioural sign of chronic pain, it would constitute an interesting way to assess animals’ pain and be a tool for further studies of the links between human patients’ attention and chronic pain.

The present study aims to evaluate domestic horses’ attentional engagement and compare it to estimates of vertebral disorders assumed to be linked to chronic pain^20^, using observations of unconditioned behaviour in their home setting. ‘Classical’ ethological observations were combined with evaluations of the state of their spine based on two approaches, one relying on a practitioner’s diagnosis, the other on static surface electromyography (sEMG), a technique considered highly reliable to evaluate human patients’ low back pains^25^. These approaches proved useful for earlier studies and led to similar evaluations for horses^20^,^21^, whose back disorders are difficult to evaluate objectively in the field by radiographic, ultrasonic or scintigraphic imaging^26^.

Alertness or attentional engagement can be affected by altered welfare^27^,^28^, but has never been measured precisely and never been related to chronic disorders such as back disorders. Here we focused on ‘observation or monitoring behaviour’ which is the most widely used indicator of attention in a variety of species and contexts (*e.g.* corvids^29^,^30^, primates^31^,^32^, dolphins^33^ and horses^34^). Monitoring includes head movements to scan the environment and gazing towards the environment while the body remains immobile^33^,^30^,^34^. It also corresponds to a readiness to react to any sensory stimulation in the environment^27^,^28^. Horses’ attention or monitoring behaviour is characterized by standing motionless at the stall door, with their neck horizontal or slightly elevated, ears and neck mobile, and slowly scanning their environment by moving their head laterally or occasionally gazing for a short moment at environmental stimuli^35^ (Fig. 1). For example, Waring (2003)^35^ describes as attentive horses, subjects that frequently exhibit visual investigation of their surroundings using monocular as well as binocular vision. Horses rotate the head and eyes looking around and often fix its gaze on nearby objects (p91) or expressions of forward attention are characterized by anteriorly directed orientation for reception of visual, auditory, olfactory, and sometimes tactile cues. The ears are up and rotated forward. The eyes are directed forward and appear to emphasize the binocular visual field. The neck and head angle adjusts to facilitate use of the sensory receptors (p274). This quiet state differs significantly from ‘vigilance’ postures, where neck and tail are raised, fixed neck, ear and head postures and that indicate strong emotional states^36^,^37^. More than 100 riding-stable horses were observed in this study. The results evidence that not only the presence/absence of back disorders is related to lower attention paid to the environment but also that attention and back disorders are correlated.

## Results

Our two studies corresponded to two different types of spine evaluations. Study 1 included 59 riding-stable horses (from 3 facilities), and study 2 included 44 horses (from 5 facilities). Instantaneous scan samples (total of 90 scans per horse) of all horses were recorded during three sessions during calm moments in the stables (at least 1h30 after riding activity for each subject) and their spines were examined independently by either a practitioner (study 1) or by sEMG (study 2) on horses’ resting day, at least 10 h after work. Spine states were evaluated by the number of vertebral sites showing an elevated tension during manual palpation or a high muscular activity during sEMG examinations (>10 mV see^21^).

### Study 1

**Figure 1:**
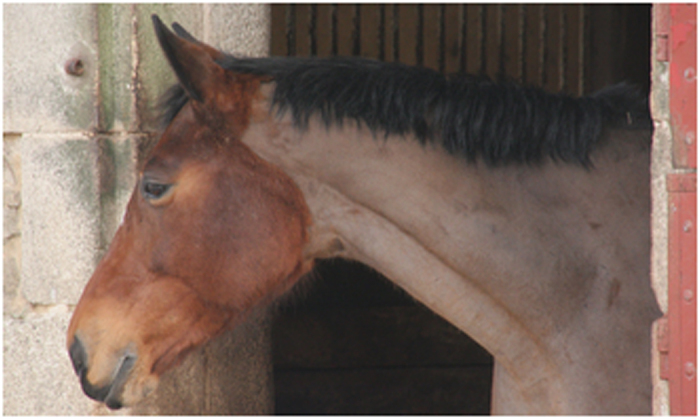
Attention or monitoring behaviour in horses is characterized by standing motionless at the stall door, with their neck horizontal or slightly elevated, ears and neck mobile, and slowly scanning their environment by moving their head laterally or occasionally gazing for a short moment at environmental stimuli.

All horses expressed monitoring behaviour at least once (Fig. 1), accounting for 21.04 ± 2.73% of their time budget. Inter-individual variations were large, ranging from 3 to 51% of the time budget spent monitoring. Time spent monitoring was explained neither by sex (Mann Whitney U test, N_female_ = 15, N_male_ = 44, 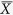
_female_ ± s.e.m. = 22.30 ± 2.47%, 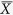
_male_ ± s.e.m = 20.61 ± 1.42%, U = 309.5, P = 0.72), nor by age (Spearman correlation test, N = 59, rs = −0.16, P = 0.22).

Spine evaluations presented large inter-individual variations ranging from 0% (for 15% of the horses) to 88% of the vertebral sites being estimated affected. This was not explained by sex (Mann Whitney U test, N_female_ = 15, N_male_ = 44, 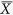
_female_ ± s.e.m = 19.48 ± 4.67%, 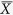
_male_ ± s.e.m = 14.30 ± 2.82%, U = 522.5, P = 0.21) and age (Spearman correlation test, N = 59, rs = 0.21, P = 0.09).

**Figure 2:**
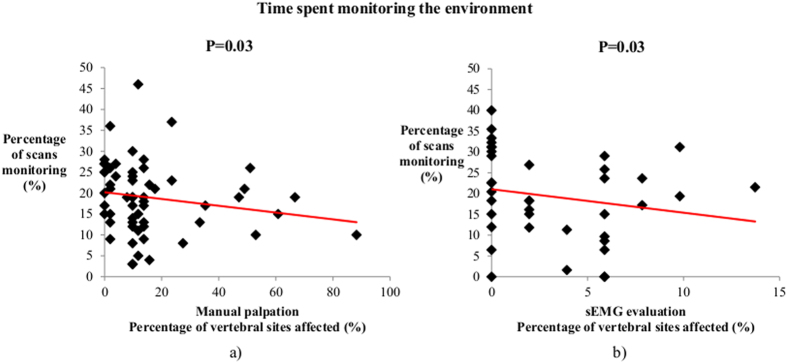
Relationship between vertebral states and monitoring behaviours. Correlations between the percentage of vertebral sites affected as indicated by (**a**) manual palpations; (**b**) sEMG evaluations; and monitoring behaviours under everyday living conditions (Spearman correlation test, p < 0.05).

We evidenced a negative correlation between behaviour and spine evaluation: the more the horses monitored their environment, the less affected they were (Spearman correlation test, N = 59, rs = −0.26, P = 0.03) (Fig. 2a). In other words, healthy horses were more attentive to their environment. Not- or slightly-affected (one vertebral site) horses monitored their environment significantly more than did severely affected (2 or more vertebral sites) horses (Mann Whitney U test, N_0−1_ = 16, N_2_ = 43, 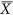
_0−1_ ± s.e.m = 17.77 ± 1.34%, 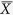
_2_ ± s.e.m = 22.06 ± 1.73%, U = 223, P = 0.03). This was not due to a restricted mobility as there was no correlation between spine evaluation and the time spent moving in the box (*i.e.* walk) (Spearman correlation test, N = 59, rs = −0.09, P = 0.49).

### Study 2

In this study, 93% of horses (41/44) expressed monitoring behaviour at least once (Fig. 1), accounting for about 20.16 ± 2.71% of their time budget. Inter-individual variations were large, ranging from 1.61 to 40.00% of the time budget spent monitoring the environment. Time spent monitoring was explained neither by sex (Mann Whitney U test, N_female_ = 22, N_male_ = 22, 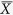
_female_ ± s.e.m = 21.79 ± 2.30%, 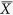
_male_ ± s.e.m = 18.55 ± 2.39%, U = 208, P = 0.43) nor by age (Spearman correlation test, N = 44, rs = 0.16, P = 0.29).

The sEMG evaluations indicated that 61.37% (N = 27) of the horses presented high muscular activity at the level of at least 1 vertebral site (from 0% to 13.72%‘ of the vertebral sites were affected per horse). As in study 1, neither sex (Mann Whitney U test, N_female_ = 22, N_male_ = 22, 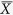
_female_ ± s.e.m = 2.67 ± 0.71%, 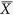
_male_ ± s.e.m = 4.28 ± 0.81%, U = 480, P = 0.73) nor age (Spearman correlation test, N = 44, rs = 0.01, P = 0.93) had any significant effect on sEMG measures.

**Figure 3:**
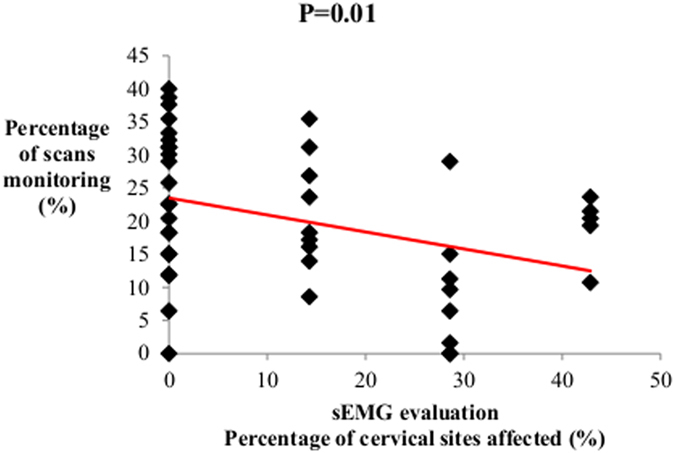
Relationship between cervical states and monitoring behaviours. Correlations between the percentage of cervical vertebral sites affected as indicated by sEMG evaluations and monitoring behaviours under everyday living conditions (Spearman correlation test, p < 0.05).

We evidenced a negative correlation between the time spent monitoring the environment and the number of vertebral sites affected according to the sEMG evaluations (Spearman correlation test, N = 44, rs = −0.35, P = 0.03) (Fig. 2b). This was confirmed by the fact that the subjects presenting mild mean muscular activity (<10 μV) expressed more monitoring behaviours than did subjects with high mean muscular activity (>10 μV) (Mann-Whitney U-test, N_lma_ = 16; N_hma_ = 22; 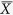
_lma_ = ± s.e.m = 25.42 ± 1.60%, 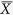
_hma_ = ± s.e.m = 14.71 ± 1.63%, U = 72, P = 0.001). Correlation between cervical spine area evaluations and monitoring was still stronger (Spearman correlation test, N = 44, rs = −0.37, p = 0.01) (Fig. 3), suggesting that neck problems may impact more attentional engagement. No significant correlations could be evidenced between spine evaluation and the time spent moving in the box (*i.e.* walk) (Spearman correlation test, N = 44, rs = −0.21, P = 0.16).

### Comparisons between studies

The rates of monitoring did not vary significantly between riding stables in both studies despite management differences (see methods) (Mann-Whitney U-test, N_study1_ = 59; N_study2_ = 44; 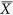
_study1_ = ± s.e.m = 21.04 ± 2.22%, 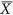
_study2_ = ± s.e.m = 20.16 ± 2.71%, U = 1056, P = 0.63). Prevalence of back disorders was higher in study 1; this could be due to management differences. Nevertheless, pooled data for the two studies revealed a still stronger negative relationship between times horses spent monitoring and the numbers of vertebral sites affected (Spearman correlation test, N = 103, rs = −0.29, P = 0.003). This correlation was confirmed when only the cervical area was considered (Spearman correlation test, N = 103, rs = −0.21, P = 0.03).

## Discussion

This study of a large sample of domestic horses observed in their home environment indicated that lower attentional engagement is correlated with the level of back disorders. This is especially clear as two different evaluation techniques used to evaluate the state of the spines gave similar results. One study showed that data for the neck area in particular contributed to this correlation. This is to our knowledge the first ‘ecological’ estimate of attention deficits related to back disorders and hence to potential chronic back pain.

However, our evaluations may not be indicative of chronic pain, as they did not measure it directly. Although animals’ chronic back pain, unless intense enough to induce immobility or lameness, is difficult to detect, all practitioners consider that elevated tenseness at vertebral sites reflects chronic pain (*e.g.*^18^,^23^). Horses’ chronic back pain is known to be correlated with aggressiveness towards humans, a behaviour often associated with illness and acute as well as chronic pain^20^,^28^,^38^.

Our demonstration showing that an animal spontaneous attention to the environment can be impaired by chronic back pain opens new lines of research for humans. It suggests that novel animal models could prove useful for preclinical research on chronic pain related cognitive impairments. Similar studies could thus provide insights into the onset of these deficits and their development or help evaluate the effects of drugs or physical exercises in the home environment. These results also promote the idea that more ethological studies in the home environment of human patients would be a way to a better understanding of the processes involved: clinical settings may be a source of stimulation that prevents direct evaluation of daily attentional engagement.

Our results are of great interest for evaluations of animal welfare and suggest that this approach should be developed for other domestic or captive species. If attentional engagement (or sensory inattention) proves a reliable indicator of chronic pain or discomfort for a variety of species, it would be a useful tool for a rapid and economical identification of potentially suffering individuals, leading to appropriate treatments or remediation. Depressed-like animals and anaemic horses tend to isolate themselves from environmental stimuli^28^,^39^,^40^, suggesting that, like human patients suffering from chronic pain, ‘they live in an impoverished and restricted environment’^17^. Chronic pain, being inescapable, is often associated with depression also in humans (*e.g.*^1^). Animal models should help disentangle the processes involved. The finding that the neck area of the spine is particularly involved in cognitive impairments related to both horses’ (this study) and humans’^11^ chronic pain deepens the parallels.

## Methods

### Ethical note

This study was approved by the University of Rennes Animal Care Committee and complied with the French laws related to animal experimentation and the European directive 86/609/CEE. Horse husbandry and care were under management of the riding stable: the horses used in this experiment were not research animals.

### Behavioural observations

Observations of horses were recorded by a single trained observer for each studies (study 1: CF, study 2: CL, trained together until they reached 98% agreement) in their individual home stall, using scan sampling^41^ every 2 minutes for 1 hour periods, repeated 3 times: first, early in the morning before the horses’ meal and riding activity; second, during the morning at least one hour after riding activity; and lastly, at the beginning of the afternoon, after having been fed and before riding activity, at least 1h30 after the morning riding activity. This yielded 90 scans per subject.

The observer walked silently and regularly along the corridor and observations were made 3 m from the horses. All behaviours were recorded (see^35^ for a detailed ethogram). Particular interest was given to their monitoring behaviours that is characterized by the horse standing motionless at the stall door, with its neck held horizontally or slightly elevated, ears and neck mobile, and slowly scanning with lateral head movements or occasional short gazes at environmental stimuli^35^. This quiet state differs highly from ‘vigilance’ postures, where neck and tail are raised and neck, ears and head in a fixed position, that indicate strong emotional states^36^,^37^.

### Study 1

The first study included 59 horses (44 geldings, 15 mares; 5–20 years old (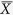 ± s.e.m = 12.81 ± 0.46); mostly French saddlebreds (68%). They were in three riding stables belonging to agricultural colleges presenting similar activities and housing conditions. These horses worked from Monday to Saturday (±4 h/day) and were free on Sundays, with maximal activity during school time (Monday to Friday). Horses were kept singly in straw-bedded individual boxes with windows facing indoors (*i.e.* onto the barn corridor) or outdoors. The boxes were cleaned once a day; horses were fed industrial pellets three times a day, hay once a day and had water *ad libitum*.

The horses’ backs were evaluated *via* manual palpation known to be efficient in detecting back pains^42^,^43^, by a licensed chiropractor who was totally blind to the results of the observations recorded before and did not know the horses beforehand. A second practitioner double checked with 94.28 ± 3.69% % agreement. Manual palpation was performed from head to tail and the mobility of each vertebral site was evaluated (N = 51 vertebral sites: 7 cervical, 18 thoracic, 6 lumbar, 5 sacral and 15 coccygeal). Examination was based on bony and soft tissue manual palpation to localise regions of vertebral stiffness based on spinal mobilisation and palpable areas of muscle hypertonicity^44^, leading to an overview of the percentage of affected vertebral sites. Examinations were performed outside the horses’ working times (horses’ backs were evaluated at least 10 h after work), in their box. The examined horse was restrained slightly by one unknown (to the horses) experimenter (MH) also blind to the behavioural observations.

### Study 2

The second study included 44 horses (22 mares, 22 geldings), of varied ages (6–23 years old; 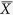 ± s.e.m. = 13.34 ± 1.79), and breeds (N = 13, mostly unregistered horses: 28.57%, French ponies: 25.00%, French Saddlebreds: 17.85% and smaller proportions of Connemaras, Anglo-Arabians, Haflingers, Merens, French trotters, Throughbreds, Welsh ponies and Pottoks) at five riding stables all over France. These horses worked two days a week, on Wednesdays and Saturdays (±4 h/day), and one to two hours a day during the rest of the week. All had at least one free day (usually Sunday). These horses were under the management of the riding stables, lived in straw-bedded individual boxes in large barns with windows facing indoors and outdoors. Most of them were fed industrial pellets (87.3%), two (43.8%) or three times (48.3%) a day. Most of them also had hay for 1 to 5 meals a day. All horses had water *ad libitum*.

These horses’ back problems were evaluated by a new approach, by sEMG, which is easily transportable, adapted to horses and has proved efficient in detecting humans’ back pain^25^. The sEMG examinations were conducted by the same experimenter (CL), using a wire free device (Myovision®). The device was composed of 2 joysticks with 5 electrodes on each, designed to record muscle activities at the level of the vertebrae at the front and at the back of the joystick location. Muscular activities recorded were sent to a receptor connected to a computer. The joysticks were placed at different levels of the vertebrae on both sides of the spine and the muscular activities at these levels were recorded (see^21^,^24^). Raw sEMG values were used (μV). As sEMG values are known to correlate with vertebral disorders^21^, a vertebral site was considered as ‘affected’ when muscular activity was above 10 μV on both sides of the spine. This 10 μV threshold is known to be related to chronic vertebral disorders as evaluated by the practitioner (Lesimple *et al.*, 2012)^21^. Back examinations were performed on the resting day, at least 10 h after work. Examinations were performed on flat ground, in the stable corridor in front of each horse’s box, without any noise or disturbance (*e.g.* working activity, people around) to avoid any intrusive muscular mobilization. The experimenter paid attention to the positions of the horses’ feet: anterior and posterior feet were aligned. Horses were kept motionless, slightly restrained.

### Statistical analyses

The numbers of subjects for each factor (site, sex, age) were unbalanced because of the availability of the different categories in the riding stables. As data were not normally distributed, we used non-parametric statistical tests^45^. Mann-Whithney U-tests were used to assess the effects of sex and vertebral state on monitoring behaviour. Spearman correlation tests investigated the relationships between horses’ ages and behavioural and vertebral variables, and the relationship between horses’ monitoring and vertebral states. Furthermore, in one riding stable where horses were kept in stalls with and without windows facing outwards, a negative correlation was found between the percentage of scans expressing monitoring behaviours and the numbers of vertebral sites affected as indicated by the sEMG evaluation (Spearman correlation test, N = 6, rs = −0.84, P = 0.03), suggesting that facing indoors or outdoors did not influence significantly this relation between back disorders and monitoring rates. All statistics were computed using R software (using an alpha of 0.05, and two-tailed tests). Descriptive statistics are means (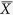) followed by standard error (s.e.m.).

## Additional Information

**How to cite this article**: Rochais, C. *et al.* Lower attention to daily environment: a novel cue for detecting chronic horses’ back pain? *Sci. Rep.* **6**, 20117; doi: 10.1038/srep20117 (2016).
